# Multiethnic prevalence of the *APOL1* G1 and G2 variants among the Israeli dialysis population

**DOI:** 10.1093/ckj/sfae397

**Published:** 2024-12-06

**Authors:** Dror Ben-Ruby, Danit Atias-Varon, Maayan Kagan, Guy Chowers, Omer Shlomovitz, Keren Slabodnik-Kaner, Neta Mano, Shany Avayou, Yariv Atsmony, Dana Levin, Edo Dotan, Ronit Calderon-Margalit, Alla Shnaider, Yosef S Haviv, Ohad S Birk, Noam Hadar, Yair Anikster, Noa Berar Yanay, Gil Chernin, Etty Kruzel-Davila, Pazit Beckerman, Benaya Rozen-Zvi, Gabriel T Doctor, Horia C Stanescu, Revital Shemer, Elon Pras, Haike Reznik-Wolf, Ayelet Hashahar Nahum, Dan Dominissini, Karl Skorecki, Asaf Vivante

**Affiliations:** Genetic Kidney Disease Research Laboratory, Sheba Medical Center, Tel-Hashomer, Ramat-Gan, Israel; Faculty of Medical and Health Sciences, Tel-Aviv University, Tel-Aviv, Israel; Genetic Kidney Disease Research Laboratory, Sheba Medical Center, Tel-Hashomer, Ramat-Gan, Israel; Department of Pediatrics B, Edmond and Lily Safra Children's Hospital, Sheba Medical Center, Tel-Hashomer, Ramat-Gan, Israel; Genetic Kidney Disease Research Laboratory, Sheba Medical Center, Tel-Hashomer, Ramat-Gan, Israel; Faculty of Medical and Health Sciences, Tel-Aviv University, Tel-Aviv, Israel; Department of Pediatrics B, Edmond and Lily Safra Children's Hospital, Sheba Medical Center, Tel-Hashomer, Ramat-Gan, Israel; Genetic Kidney Disease Research Laboratory, Sheba Medical Center, Tel-Hashomer, Ramat-Gan, Israel; Faculty of Medical and Health Sciences, Tel-Aviv University, Tel-Aviv, Israel; Department of Pediatrics B, Edmond and Lily Safra Children's Hospital, Sheba Medical Center, Tel-Hashomer, Ramat-Gan, Israel; Genetic Kidney Disease Research Laboratory, Sheba Medical Center, Tel-Hashomer, Ramat-Gan, Israel; Faculty of Medical and Health Sciences, Tel-Aviv University, Tel-Aviv, Israel; Department of Pediatrics B, Edmond and Lily Safra Children's Hospital, Sheba Medical Center, Tel-Hashomer, Ramat-Gan, Israel; Genetic Kidney Disease Research Laboratory, Sheba Medical Center, Tel-Hashomer, Ramat-Gan, Israel; Department of Pediatrics B, Edmond and Lily Safra Children's Hospital, Sheba Medical Center, Tel-Hashomer, Ramat-Gan, Israel; Genetic Kidney Disease Research Laboratory, Sheba Medical Center, Tel-Hashomer, Ramat-Gan, Israel; Faculty of Medical and Health Sciences, Tel-Aviv University, Tel-Aviv, Israel; Arrow Project, Sheba Medical Center, Tel-Hashomer, Ramat-Gan, Israel; Genetic Kidney Disease Research Laboratory, Sheba Medical Center, Tel-Hashomer, Ramat-Gan, Israel; Genetic Kidney Disease Research Laboratory, Sheba Medical Center, Tel-Hashomer, Ramat-Gan, Israel; Faculty of Medical and Health Sciences, Tel-Aviv University, Tel-Aviv, Israel; Department of Pediatrics B, Edmond and Lily Safra Children's Hospital, Sheba Medical Center, Tel-Hashomer, Ramat-Gan, Israel; Genetic Kidney Disease Research Laboratory, Sheba Medical Center, Tel-Hashomer, Ramat-Gan, Israel; The George S. Wise Faculty of Life Sciences, Tel-Aviv University, Tel-Aviv, Israel; The Taub Faculty of Computer Science, Technion Israel Institute of Technology, Haifa, Israel; Braun School of Public Health, Hadassah Medical Center, Faculty of Medicine, Hebrew University, Jerusalem, Israel; Department of Nephrology, Soroka University Medical Center, Beer-Sheva, Israel; Faculty of Health Sciences, Ben Gurion University, Beer-Sheva, Israel; Department of Nephrology, Soroka University Medical Center, Beer-Sheva, Israel; Faculty of Health Sciences, Ben Gurion University, Beer-Sheva, Israel; Faculty of Health Sciences, Ben Gurion University, Beer-Sheva, Israel; Genetics Institute at Soroka Medical Center, Beer-Sheva, Israel; Faculty of Health Sciences, Ben Gurion University, Beer-Sheva, Israel; Faculty of Medical and Health Sciences, Tel-Aviv University, Tel-Aviv, Israel; Metabolic Diseases Unit, Edmond and Lily Safra Children's Hospital, Sheba Medical Center, Tel-Hashomer, Ramat-Gan, Israel; Nephrology Department, Hillel Yaffe Medical Center, Hadera, Israel; The Ruth and Bruce Rappaport Faculty of Medicine, Technion Israel Institute of Technology, Haifa, Israel; Department of Nephrology and Hypertension, Kaplan Medical Center, Faculty of Medicine, Hebrew University of Jerusalem, Rehovot, Israel; Nephrology Department, Galilee Medical Center, Nahariya, Israel; The Azrieli Faculty of Medicine, Bar-Ilan University, Safed, Israel; Faculty of Medical and Health Sciences, Tel-Aviv University, Tel-Aviv, Israel; Institute of Nephrology and Hypertension, Sheba Medical Center, Tel-Hashomer, Ramat-Gan, Israel; Faculty of Medical and Health Sciences, Tel-Aviv University, Tel-Aviv, Israel; Department of Nephrology and Hypertension, Rabin Medical Center, Petah Tikva, Israel; Centre for Genetics and Genomics, Department of Renal Medicine, UCL Division of Medicine, University College London, London, UK; Centre for Genetics and Genomics, Department of Renal Medicine, UCL Division of Medicine, University College London, London, UK; The Ruth and Bruce Rappaport Faculty of Medicine, Technion Israel Institute of Technology, Haifa, Israel; Faculty of Medical and Health Sciences, Tel-Aviv University, Tel-Aviv, Israel; The Danek Gertner Institute of Human Genetics, Sheba Medical Center, Tel-Hashomer, Ramat-Gan, Israel; Faculty of Medical and Health Sciences, Tel-Aviv University, Tel-Aviv, Israel; The Danek Gertner Institute of Human Genetics, Sheba Medical Center, Tel-Hashomer, Ramat-Gan, Israel; The Danek Gertner Institute of Human Genetics, Sheba Medical Center, Tel-Hashomer, Ramat-Gan, Israel; Faculty of Medical and Health Sciences, Tel-Aviv University, Tel-Aviv, Israel; Institute of Hematology, Sheba Medical Center, Tel-Hashomer, Ramat-Gan, Israel; Cancer Research Center, Sheba Medical Center, Tel-Hashomer, Ramat-Gan, Israel; The Ruth and Bruce Rappaport Faculty of Medicine, Technion Israel Institute of Technology, Haifa, Israel; The Azrieli Faculty of Medicine, Bar-Ilan University, Safed, Israel; Rambam Health Care Campus, Haifa, Israel; Genetic Kidney Disease Research Laboratory, Sheba Medical Center, Tel-Hashomer, Ramat-Gan, Israel; Faculty of Medical and Health Sciences, Tel-Aviv University, Tel-Aviv, Israel; Department of Pediatrics B, Edmond and Lily Safra Children's Hospital, Sheba Medical Center, Tel-Hashomer, Ramat-Gan, Israel; Pediatric Nephrology Unit, Edmond and Lily Safra Children's Hospital, Sheba Medical Center, Tel-Hashomer, Ramat-Gan, Israel

**Keywords:** chronic renal failure, CKD, ethnicity, gene polymorphism, podocytes

## Abstract

**Background and hypothesis:**

The two apolipoprotein L1 (*APOL1*) variants, G1 and G2, are common in populations of sub-Saharan African ancestry. Individuals with two of these alleles (G1 or G2) have an increased risk for a spectrum of non-diabetic chronic kidney diseases. However, these variants are typically not observed outside of populations that self-identify as current continental Africans or having clear recent African ancestry such as, most notably, African Americans, and other large population groups in the Americas and several European countries. We hypothesized that the diverse ethnic groups within the Israeli population may exhibit varying levels of recent African ancestry. Therefore, it is plausible that *APOL1* risk alleles might be present even in individuals who do not self-identify as being of sub-Saharan African descent.

**Methods:**

We non-selectively screened people with kidney failure across Israel for *APOL1* risk variants using restriction fragment length polymorphism.

**Results:**

We recruited 1744 individuals from 38 dialysis units in Israel. We identified eight patients of Moroccan Jewish, Bedouin, or Muslim Arab ancestry, who carry at least one G1 or G2 allele. None of the eight patients carried the protective *APOL1* p.N264K variant. Furthermore, despite all Bedouin individuals being G2 heterozygous, the G2 minor allele frequency was significantly enriched in kidney failure cases compared to ethnically matched controls (*P* = .006).

**Conclusions:**

These findings show that *APOL1* G1 and G2 allelic variants are present in populations previously not appreciated to possess recent sub-Saharan ancestry and suggest that a single G2 risk variant may confer increased risk for chronic kidney disease in certain population contexts.

## Graphical Abstract

**Graphical Abstract:**
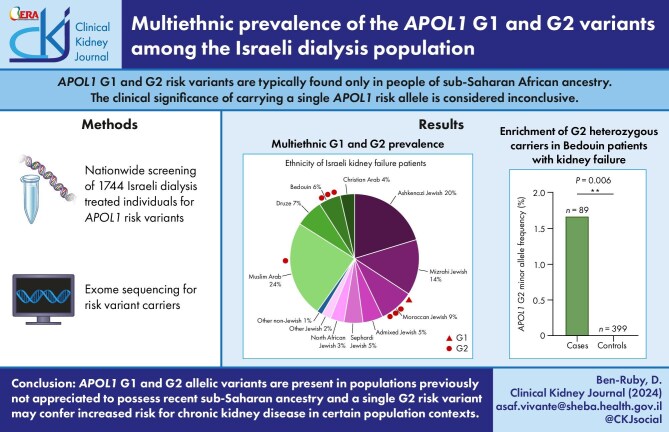

KEY LEARNING POINTS **What was known:**

- *APOL1* G1 and G2 risk variants are typically found only in people identifying as being of sub-Saharan African ancestry.
- These variants are known to increase the risk of chronic kidney diseases in a recessive inheritance mode.
- The clinical significance of carrying a single *APOL1* risk allele is considered inconclusive.

**This study adds:**

- We identified these variants in Israeli individuals with kidney failure of Bedouin, Muslim Arab, and Moroccan Jewish ethnicity.
- In the Bedouin population, a single G2 allele was associated with increased risk for kidney disease.

**Potential impact:**

- This study highlights the presence of *APOL1* risk alleles in several Middle-Eastern populations who do not self-identify as being of sub-Saharan African ancestry, and suggests that a single G2 risk variant may confer increased risk for chronic kidney disease in certain population contexts.

## INTRODUCTION

Kidney failure is four times more common in populations of recent sub-Saharan African ancestry, compared to populations without such ancestry [1]. A significant component of this difference has been attributed to G1 and G2 variants in apolipoprotein L1 (*APOL1*) [2, 3], which confer an increased risk for a spectrum of non-diabetic chronic kidney diseases (CKD) such as hypertension-related kidney disease, focal segmental glomerulosclerosis (FSGS), human immunodeficiency virus associated nephropathy, and progression of lupus nephritis, currently collectively referred to as APOL1-mediated kidney disease (AMKD) [4, 5].

The ancestral versions of *APOL1* are circulatory proteins that protect human and some non-human primates against *Trypanosoma brucei brucei* and probably other pathogens [6]. The kidney disease risk variants, *APOL1*-derived G1 and G2, rose to high frequency in Western and Central Africa in response to selective pressure from the emergence of Trypanosoma species with extended virulence (*Trypanosoma brucei gambiense* and *rhodesiense*) [7]. These species developed resistance to the ancestral *APOL1* (G0), driving the evolutionary advantage of the G1 and G2 variants. The G2 variant has been shown to reduce the risk of infection by *T. b. rhodesiense*, prevalent in East Africa, while the G1 variant decreases the risk of severe trypanosomiasis caused by *T. b. gambiense*, common in West Africa [8]. This protective effect led to positive selective pressure on these variants. These events likely occurred ∼10 000 years ago, after the early emigration waves to other continents (20 000–100 000 years ago or even earlier), and were therefore likely contained in that region for many years [9, 10]. It is believed that they were only carried to the Americas during the sixteenth to nineteenth centuries, during the forced relocation brought about with the brutal trans-Atlantic slave trade [11]. Consequently, most *APOL1* studies have focused on people self-reporting as Africans or African Americans. However, other populations who share recent African ancestry (e.g. Latino) may also harbor *APOL1* risk variants, which calls for research on the basis of genetic ancestry, rather than self-reported ethnic groups [7]. When examining the haplotype neighboring the G1 and G2 loci, defined by three amino acids (p.150, p.228, p.255), G1 and G2 are in near-complete linkage disequilibrium with the EIK haplotype variants (glutamic acid, E isoleucine, I; lysine, K), predominantly found in sub-Saharan Africa. By contrast, the G0 variant is found on multiple haplotypes, including EIK, KIK (lysine, K; isoleucine, I; lysine, K), and EMR (glutamic acid, E; methionine, M; arginine, R), common in East Asian and European populations [12]. This suggests that each *APOL1* risk variant originated from a single founder event in sub-Saharan Africa.

The Israeli population is a tapestry comprising numerous population groups, many representing recent origins in multiple continents. Approximately 80% of the country's population are Jewish Israelis, and 20% are Arab Israelis. Over the past 75 years, the Jewish Israeli population has increased >10-fold from 650 000 in 1948 to >7 000 000 today, due to substantial immigration as well as births. The current Israeli Jewish population comprises many population groups, including Ashkenazi Jews, primarily of European ancestry; Sephardi Jews, originally from the Iberian Peninsula who migrated mainly to the Balkan and Turkey following expulsion in the fifteenth century; North-African Jews, of both Iberian and Berber ancestries; Mizrahi Jews, from the Middle East, Central-Asia, and the Indian subcontinent; and other smaller distinct Jewish populations [13]. Population genetics studies have shown that despite the geographic distance, many but not all members of these diverse communities demonstrate greater degrees of shared ancestry with each other than do the non-Jewish populations in their recent ancestral regions. This shared ancestry component, dating back several millennia, has a Levant origin signal [14, 15].

The Arab Israeli population whose increase is mostly attributed to high birth rates, comprises multiple ethnicities based on both religious and cultural practice and geographic origin. These include: Sunni Muslims, descendants of the Arabian tribes of the Islamic expansions [16]; Orthodox, Catholic, or Protestant Christians; Druze (a transnational isolate) [17], and Bedouins (traditional nomadic tribal society) [18]. These populations typically exhibit high levels of endogamy, and varying rates of consanguineous marriages [19].

The CKD incidence in Israel stands out as one of the highest in the world [20], especially among Arab Israelis [21, 22]. We hypothesized that the diverse ethnic groups within the Israeli population may exhibit varying levels of recent African ancestry. Therefore, it is plausible that *APOL1* risk alleles might be present even in individuals who do not self-identify as being of sub-Saharan African descent. Consequently, our aim in this study was to investigate the prevalence of *APOL1* risk variants among Israeli patients with kidney failure.

## MATERIALS AND METHODS

### Study participants

We conducted a nationwide multicenter prospective study of all Israeli dialysis units. The study population included adults of all ages who underwent maintenance dialysis. After obtaining informed consent, clinical and pedigree data were collected from the patients and their primary nephrologists via a standardized questionnaire [23, 24]. Subsequently, blood samples were collected from participating individuals, as well as from additional family members if available. The study was approved by the Institutional Review Boards of Sheba Medical Center and the Israeli Ministry of Health, as well as by the corresponding institutional review boards at the other participating centers, and was conducted in adherence to the principles outlined in the Declaration of Helsinki. Ethnically matched controls were obtained from the genetic institutes of Sheba and Soroka Medical Centers.

### Clinical assessment and ethnicity classification

At the time of study recruitment, all participants underwent a clinical interview including a comprehensive review of their medical files, retrieval of detailed previous medical history, imaging studies, and kidney biopsy reports when available. Furthermore, information regarding family medical history, consanguinity, ethnicity, and timing of kidney failure were obtained. Subjects were assigned with a primary clinical diagnosis based on their medical records. The ethnicity of each subject was recorded at the time of recruitment by self-identification. For Jewish participants, ethnicity was further classified according to the population groupings described in the Introduction section.

### *APOL1* genotyping

#### Restriction fragment length polymorphism (RFLP)

We used two restriction enzymes, HindIII and MluCI, to screen for the presence of *APOL1* G1 and G2 variants. SNP rs73885319 [A>G, G1 (G)] cancels a recognition site of endonuclease HindIII. SNP rs71785313 (6-bp deletion, G2) cancels a recognition site of endonuclease MluCI. Thus, to confirm and screen for the mutations, we PCR amplified a 539 bp fragment as previously described [2]. The resulting amplicons were simultaneously digested with both endonucleases, HindIII and MluCI (New England Biolabs, R3104 and R0538). Amplicons from the G0 allele were digested into 41, 85, 138, and 275 bp fragments, while those from the G1 and G2 alleles were digested into 41, 85, and 413 bp and 41, 217, and 275 bp fragments, respectively, and identified using gel electrophoresis (MetaPhor Agarose, Thermo Fisher Scientific). Sanger sequencing was employed to confirm the detection of risk variants identified by RFLP and to verify the presence of the second missense substitution comprising the G1 variant, rs60910145 [T > G, G1 (M)].

#### PCR cloning

The previously mentioned PCR amplicons were cloned into p.CR2.1 V Vector using the TA Cloning Kit (Invitrogen, 45-0046), and transformed into competent *E. coli* (Zymo Research, T3001). Plasmids were harvested using the GeneJET Plasmid Miniprep Kit (Thermo Scientific, K0503), digested with EcoRI (New England Biolabs), and sequenced using standard M13 primers.

#### Exome sequencing (ES) and variant analysis

Proband ES was performed on genomic DNA for all *APOL1* risk variants carriers using an IDT xGeb Exome Hyb Panel V2 and Illumina NoveSeq6000 sequencing technology. For each sample, paired end-reads (2 × 100 bp) were obtained, processed, and mapped to the genome. The BWA-MEM algorithm (v.0.7.17-r1188) was used to align the sequence reads to the human reference genome (hg37). The HaplotypeCaller algorithm of GATK v.4.4.0.0 was applied for variant calling, as recommended in the best practice pipeline [25]. Sequencing data was uploaded to the Franklin Platform (Genoox, Tel Aviv, Israel, available at https://franklin.genoox.com), which contains 10 000 ethnically matched samples. Public databases such as the Genome Aggregation Database (gnomAD) and Greater Middle East Variome were also used for variant filtering. All rare (minor allele frequency <1%), non-synonymous and splice variants in genes present in the Online Mendelian Inheritance in Man database were assessed. Additionally, an in-house generated list of genes [24] with reported CKD-causative mutations was used as a second targeted analysis. Exome data was interpreted according to the American College of Genetic and Genomic Medicine (ACMG) guidelines [26]. The Genoox Classification Engine runs 17 of the 28 ACMG criteria automatically (PVS1, PS1, PM1, PM2, PM4, PM5, PP2, PP3, PP5, BA1, BS1, BS2, BP1, BP3, BP4, BP6, and BP7) [27]. The remaining rules cannot be automated, as they require clinical information specific to the patient genotype, e.g. familial data, *de novo* evidence (PS2, PM6), segregation data (PP1, BS4), and/or allelic data (PM3, BP2). Therefore, the remaining criteria were manually classified. Finally, variants were classified as pathogenic, likely pathogenic, variants of unknown significance, likely benign, or benign. Additionally, copy number variants (CNVs) were called using the Rainbow algorithm in the Franklin Platform by comparing the average coverage and improperly paired reads per area of a sample to data from a cohort of normal samples. Areas with significant differences relative to the cohort were considered CNVs.

### Data analysis

Data analysis was performed using the SPSS software (IBM SPSS Statistics, v.29, IBM Corporation, 2023). The Fisher's exact test was employed to evaluate the association between genotype frequencies and kidney disease risk. Specifically, we assessed whether the observed frequencies of heterozygote *APOL1* risk allele genotypes differed significantly from the expected frequencies under the null hypothesis of no association.

## RESULTS

### Study population

**Figure 1:**
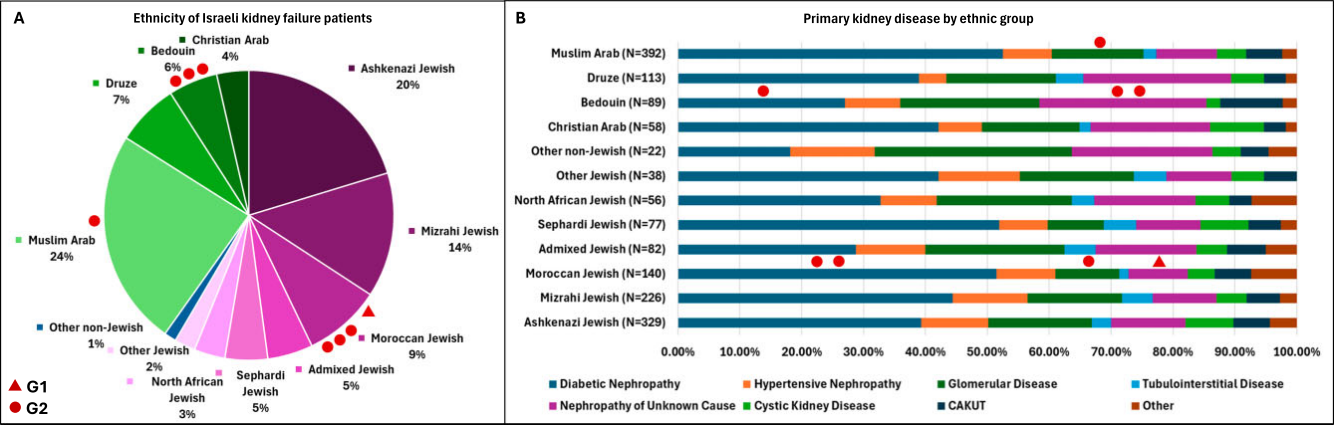
The ethnic and clinical diversity of the Israeli dialysis population. (**A**) Ethnic identification of 1744 Israeli kidney failure patients. See Methods and Introduction for the named categories of the different ethnic groups. (**B**) The proportion of cases attributed to common primary kidney diseases within each ethnic group. In both panels, the red dots indicate individuals carrying either *APOL1* G1 or G2 alleles. Ethnicity data and primary kidney disease data were not available in 6.99% and 2.92% of the cohort, respectively.

We screened 1744 people with kidney failure from 38 dialysis units evenly distributed across Israel between 2019 and 2023. These represent roughly 25% of the ∼7000 dialysis-treated patients nationwide during this period [28]. Among these, 58.5% were Jewish and 40.2% were Arab, while 1.3% were from other ethnic groups. Among the Jewish Israelis in the study, the largest population sub-group was Ashkenazi Jewish (accounting for 20.3% of all cases), followed by Mizrahi Jewish (14.0%), Moroccan Jewish (8.6%), Sephardi Jewish (4.8%), other North-African Jewish (3.5%), and Other Jewish (2.3%) (Fig. 1). Approximately 5% of the population was of admixed Jewish ancestry. The largest Arab Israeli population group was Muslim Arabs (accounting for 24.1% of all cases), followed by Druze (7.0%), Bedouin (5.5%), and Christian Arabs (3.6%) (Fig. 1).

The study cohort's primary kidney diseases were classified into eight sub-groups. The most common primary kidney disease was diabetic nephropathy (41.7%), followed by glomerular diseases (16.8%), nephropathy of unknown cause (13.4%), hypertension-related nephropathy (9.5%), congenital anomalies of the kidney and urinary tract (CAKUT, 6.4%), cystic kidney disease (5.7%), tubulointerstitial disease (3.1%), and other etiologies (3.4%).

### *APOL1* risk variant analyses

Among the study population, we detected eight unrelated patients, without known recent sub-Saharan ancestry, who harbored at least one *APOL1* risk allele (G1 or G2) (Fig. 1). Four Jewish patients of Moroccan ancestry and three Bedouin patients carried one risk allele, and one patient of Muslim Arab ancestry carried two risk alleles (Table 1). In one case where the ancestry was mixed Moroccan and Iraqi Jewish, we confirmed that the G2 allele segregated from the Moroccan Jewish parent. The clinical characteristics of these patients are presented in Table 1.

**Table 1:** Clinical characteristics of patients with kidney failure carrying at least one *APOL1* risk variant.

Family-Individual	Ancestry	*APOL1* genotype	*APOL1* haplotype^a^	*APOL1* p.264	Primary kidney phenotype	Age at kidney failure (years)	Additional clinical manifestations	Family history of CKD	Other variants detected in ES
717–21	Bedouin	G2/G0	EIK/EIK	N/N	Nephropathy of unknown cause	61			
760–21	Bedouin	G2/G0	EIK/EIK	N/N	Nephropathy of unknown cause	43	HTN, dyslipidemia		
767–21	Bedouin	G2/G0	EIK/EIK	N/N	Diabetic nephropathy	60	HTN, dyslipidemia, T2DM, gout, nephrolithiasis		
1008–21	Muslim Arab	G2/G2	EIK/EIK	N/N	FSGS	20	HTN, asthma, ichthyosis congenita	Brother, uncle, and grandmother	*CYP4F22*: c.177C>G, p.F59L hom. (ichthyosis)
1042–21	Moroccan Jewish	G2/G0	EIK/EMR	N/N	Diabetic nephropathy, hypertensive nephrosclerosis	81	T2DM, HTN	Brother	
1144–21	Moroccan Jewish	G2/G0	EIK/EIK	N/N	Diabetic nephropathy	67	T2DM, HTN, dyslipidemia, IHD, HF		
1200–21	Moroccan Jewish	G2/G0	EIK/EIK	N/N	Lupus nephritis	40	SLE, HTN		
1630–21	Moroccan Jewish	G1/G0	EIK/EIK	N/N	Nephropathy of unknown cause	61	FMF^b^, HCV		*MEFV*: c.2080A > G, p.M694V het. (FMF)

a APOL1 haplotype refers to amino acids (E, glutamic acid; I, isoleucine; K, lysine) at the protein positions 150, 228, and 255, respectively, neighboring the G1 and G2 loci.

b Although it cannot be ruled out without a biopsy, FMF-caused amyloidosis is unlikely to be the cause of 1630–21’s kidney disease, given long-term Colchicine treatment. Abbreviations: AA, amino acids; AR, autosomal recessive; FMF, familial Mediterranean fever; HCV, chronic hepatitis C virus infection; Het., heterozygous; Hom., homozygous; HTN, hypertension; HF, heart failure; IHD, ischemic heart disease; N, asparagine; SLE, systemic lupus erythematosus; T2DM, type 2 diabetes mellitus.

Subsequently, using ES, we confirmed that none of the eight patients carried additional diagnostic variant/s explaining their kidney disease (Table 1). Furthermore, none carried the protective *APOL1* p.N264K variant [29]. The neighboring *APOL1* haplotype (amino acids 150, 228 and 255) was also analyzed using TA cloning in cases of Moroccan Jewish or Bedouin ancestry. This analysis confirmed that the G2 variant in both populations exists on the EIK haplotype that is common in sub-Saharan Africa and of negligible frequency in non-African populations [12].

Finally, we screened 399 Bedouin and 456 Moroccan Jewish controls without kidney disease. One Bedouin control individual had a G1 heterozygous genotype, and the *APOL1* G2 allele was completely absent in Bedouin controls. The G2 minor allele frequency was significantly higher among Bedouins with kidney failure compared to their ethnically matched controls (1.68% vs. 0%, respectively; *P =* .006, Fisher's exact test). Despite the small number of individuals carrying each variant, we conducted a sub-analysis of the data based on clinical diagnoses: diabetic nephropathy versus non-diabetic kidney disease. The heterozygous *APOL1* G2 genotype was significantly associated with non-diabetic kidney disease (*P =* .019), but showed no association with diabetic nephropathy (*P =* .057). Being very rare in both groups, the G1 allele was not significantly associated with either cases or controls (Table 2). In the Moroccan Jewish population, the G1 and G2 minor allele frequencies were not significantly higher in people with kidney failure compared to controls (0.36% and 1.07% vs. 0.11% and 0.33%, *P =* .411 and .144, respectively). Similar data sub-analysis did not demonstrate significant differences (diabetic nephropathy cases 0% and 1.43% vs. 0.11% and 0.33%, *P =* 1 and *P =* .134; non-diabetic kidney disease 0.71% and 0.71% vs. 0.11% and 0.33%, *P =* .247 and *P =* .432, respectively).

**Table 2:** Minor allele frequency of *APOL1* risk variants in the Bedouin population.

	Minor allele frequency (%)								
	All cases			Non-diabetic kidney disease			Diabetic nephropathy		
	Kidney failure (*n* = 89)	Controls (*n* = 399)	*P* value	Kidney failure (*n* = 65)	Controls (*n* = 399)	*P* value	Kidney failure (*n* = 24)	Controls (*n* = 399)	*P* value
G1 (G) rs73885319	0/178 (0%)	1/798 (0.12%)	1	0/130 (0%)	1/798 (0.12%)	1	0/48 (0%)	1/798 (0.12%)	1
G1 (M) rs60910145	0/178 (0%)	1/798 (0.12%)	1	0/130 (0%)	1/798 (0.12%)	1	0/48 (0%)	1/798 (0.12%)	1
G2 rs71785313	3/178 (1.68%)	0/798 (0%)	**.006**	2/130 (1.53%)	0/798 (0%)	**.02**	1/48 (2.08%)	0/798 (0%)	.06
Either G1 or G2	3/178 (1.68%)	1/798 (0.12%)	**.02**	2/130(1.53%)	1/798 (0.12%)	.05	1/48 (2.08%)	1/798 (0.12%)	.11

Minor allele frequency of *APOL1* risk variants in Arab Bedouins and Moroccan Jews with kidney failure versus ethnically matched controls. Controls were collected in Soroka and Sheba Medical Centers, as well as by the National Laboratory for the Genetics of Israeli Populations at Tel Aviv University (NLGIP). *P* value is calculated for a two-sided Fisher's exact test. Significant *P* values are indicated in bold (*P* < .05).

## DISCUSSION

In this study, we prospectively screened a non-selective multiethnic cohort of 1744 Israeli individuals with kidney failure for the presence of *APOL1* G1 and G2 variants. These variants were detected in eight individuals of Bedouin, Moroccan Jewish, and Muslim Arab ancestries. In addition, in the Bedouin population, occurrence of a single G2 variant was significantly associated with kidney failure.

Our findings point to several suggestive clinically relevant insights. First, is the observation of *APOL1* risk variants in non-sub-Saharan African populations. The nationwide, multiethnic scope of this study enabled us to denote and calculate the prevalence of *APOL1* G1 and G2 variants across three distinct populations in Israel: Bedouins, Muslim Arabs, and Moroccan Jews. The absence of known sub-Saharan African ancestry in these populations, originating from various regions of North Africa and the Levant, suggests that *APOL1* risk variants may exist in additional, previously unreported populations who do not self-identify as African. These populations may still carry significant levels of sub-Saharan African ancestry. Second, is the association between one risk allele genotype and kidney failure, at least for the *APOL1* G2 risk allele in Bedouins. A kidney disease phenotype for a single risk allele has been previously suggested but has not been fully established [30–33].

This study has several limitations that should be considered. First, the study population consists of specific, regional populations, and therefore conclusions should be made with caution regarding other populations. Second, ethnicity was determined by self-identification rather than genome-wide admixture analysis, meaning some individuals may have unrecognized sub-Saharan African ancestry. However, such genetic analysis is not routinely available, making self-identification more practical in clinical settings. Third, the small number of individuals carrying *APOL1* risk alleles in each group calls for validating these results in a larger number of individuals from additional cohorts.

The spread of *APOL1* G1 and G2 from sub-Saharan African to Jewish and Arab populations is likely due to geographic proximity. Despite the absence of large documented migrations linking the Moroccan Jewish, Bedouin, or Muslim Arab populations with sub-Saharan Africa, northward and trans-Mediterranean migration from sub-Saharan Africa, as well as more recent historical admixture between the populations have occurred [34, 35]. Jewish communities, especially those of north-African origin, and other non-Jewish Levantine communities, have been shown to contain some levels of sub-Saharan admixture [14, 36]. This strengthens the claim made by Nadkarni *et al*. [7] that individuals who do not self-identify as Africans may still share important levels of African ancestry and harbor *APOL1* risk alleles. Interestingly, *APOL1* risk variants were not observed in individuals of Ethiopian Jewish ancestry, consistent with previous studies [37]. The absence of these variants in Ethiopian Jews makes the discovery of *APOL1* risk variants in Moroccan and Arabian populations even more surprising, as these populations are not typically considered to have sub-Saharan origins. However, it appears that despite not being typically appreciated, these populations do carry some levels of sub-Saharan ancestry, which may explain the presence of *APOL1* risk alleles. Furthermore, the shared ancestry of the Israeli Arab population with other regional Arab populations [14, 38] provides insight into the genetic landscape of Middle-Eastern and global Arab populations, which may also carry *APOL1* risk alleles.

An important, unresolved question is the clinical significance of carrying a single *APOL1* risk allele, which is currently considered inconclusive [5, 39, 40]. However, in the Bedouin population, a single G2 allele appears to confer an increased risk of kidney disease. Several factors may explain the observation of a significant heterozygote risk in this population. First, this study focused on cases with the extreme phenotype of kidney failure, rather than the broader population encompassing all stages of CKD. This focus may have allowed us to detect risk association signals that would otherwise be too nuanced to detect in a much larger population in which early stages of CKD are not consistently identified.

Second, the endogamous nature and high consanguinity rate in the Bedouin population may be important factors. Sub-Saharan African populations, with their large long-term population sizes and deep population divergence times, exhibit some of the highest genetic diversity in the world [41]. By contrast, the Bedouin population has a very high consanguinity rate (44%) and an average inbreeding coefficient of 0.0238 (for context, the offspring of first-cousin mating would have an inbreeding coefficient of 0.0625) [19, 42]. The high genome-wide heterozygosity rate in African populations is possibly protective. By contrast, the high genome-wide homozygosity rate in the Bedouin population may increase the penetrance of *APOL1* risk alleles. Such inbred, endogamous populations may act as human knockouts for yet-to-be-identified genomic loci that confer protection from AMKD.

A third factor may be the higher proportion of recent European ancestry in Bedouins compared to other populations [14]. It has been shown that a lower proportion of African ancestry among individuals with the *APOL1* high-risk genotype may be associated with an increased risk of kidney disease. This effect is seen in Hispanics, who face higher risks despite lower frequencies of the risk alleles of *APOL1* risk alleles and risk alleles in strong linkage disequilibrium with *APOL1* [30, 33, 43]. Similarly, a higher proportion of European ancestry in the context of *APOL1* high-risk genotype is associated with a greater risk of FSGS [44]. These findings suggest the possibility of an unidentified protective variant within the African genetic background that might mitigate the deleterious effects of *APOL1*, similar to the *APOL1* p.N264K variant [29]. Therefore, we hypothesize that the affected Bedouin population may harbor a higher proportion of recent European ancestry, potentially mediating increased CKD risk associated with even a single *APOL1* G2 allele.

Another important, unresolved question is the mechanism by which the two *APOL1* risk variants cause kidney disease. Numerous studies have shown that *APOL1* G1 and G2 cause kidney disease by what appears to be a recessive gain-of-function mechanism [45–47]. Two models have been suggested to explain this rare mechanism and have been discussed at length elsewhere [48–50]: the “G0 rescue” model and the “toxic threshold” model. Briefly, the G0 rescue model suggests that the G0 allele is protective and counteracts the toxic effect of G1 and G2, thus preventing kidney disease risk in the heterozygote genotype. The toxic threshold model suggests that there is a certain threshold of toxicity that must be exceeded to confer a risk of kidney disease, for which one risk allele is not enough. While not addressing this question directly, this study provides modest evidence against the G0 rescue model by showing significant kidney disease risk in Bedouin heterozygous G2 carriers.

In conclusion, despite AMKD being a rare cause of kidney failure in the Israeli population, *APOL1* risk variants, G1 and G2, do exist in populations without explicit recent sub-Saharan ancestry. In such populations, one risk allele may be sufficient to confer increased risk of kidney failure. These findings could have important clinical implications in diverse, unrepresented global populations, and underscore the importance of considering the contribution of *APOL1* risk variants and an AMKD etiology in broader groups of patients, beyond those identifying as being of African descent.

## Data Availability

The original contributions presented in this study, including all relevant data and materials, are included in the manuscript. For further inquiries or additional data requests, please contact the corresponding author.
